# Associations Between Childhood Neglect and Depressive Symptoms: The Mediating Effect of Avoidant Coping

**DOI:** 10.1155/da/9959689

**Published:** 2024-11-30

**Authors:** Laura Eggert, Laura Kenntemich, Leonie von Hülsen, Jürgen Gallinat, Ingo Schäfer, Annett Lotzin

**Affiliations:** ^1^Department of Psychiatry and Psychotherapy, University Medical Center Hamburg Eppendorf, Hamburg, Germany; ^2^Department of Psychology, MSH Medical School Hamburg, Institute of Clinical Psychology and Psychotherapy, Hamburg, Germany

**Keywords:** childhood neglect, coping, COVID-19, depressive symptoms

## Abstract

**Background:** Individuals with a history of childhood neglect may be vulnerable to develop depression, as they may more often use avoidant strategies to cope with the stressors. This study examined (1) whether a history of childhood neglect was associated with higher levels of depressive symptoms and (2) whether avoidant coping behaviors mediated this association.

**Methods:** In total, *N* = 2245 German adults (mean age = 41.1 years, age range = 18–82 years, 70.2% female) were recruited from the general population between June and September 2020 during the COVID-19 pandemic. Childhood neglect (Adverse Childhood Experience Questionnaire [ACE]), depressive symptoms (Patient Health Questionnaire [PHQ-9]), and three avoidant coping behaviors (substance use, behavioral disengagement, and self-blame; Brief Coping Orientation to Problems Experienced [COPE]) were assessed. Using structural equation modeling (SEM), we examined the direct pathway from childhood neglect to depressive symptoms in a simultaneous parallel multiple mediation model and the possible mediating paths of avoidant coping behaviors.

**Results:** Childhood neglect was positively and significantly associated with depressive symptoms (*β* = 0.24, *p* < 0.01) while controlling for the presence of childhood abuse. The three avoidant coping behaviors significantly mediated this association (substance use: bias-corrected 95% confidence intervals [BC 95% CI], 0.02, 0.05; behavioral disengagement: BC 95% CI, 0.04, 0.12; and self-blame: BC 95% CI, 0.16, 0.19). Post hoc contrasts between the mediators showed that self-blame had a significantly stronger indirect effect than substance use (BC 95% CI, −0.12, −0.01).

**Conclusions:** This study provides evidence that avoidant coping behaviors mediate the association between childhood neglect and depressive symptoms in adults. Avoidance coping behaviors may be a promising target for psychological interventions to reduce depressive symptoms.

## 1. Introduction

Childhood neglect is the most common form of childhood maltreatment [1], with a prevalence of 14.8% for men and 13.9% for women in the general population [2]. Childhood abuse and neglect are recognized as important risk factors for a variety of mental health problems, such as depression, across the lifespan (for meta-analyses, see [3, 4]). However, most research has focused on childhood abuse or has grouped neglect and abuse together, while the specific role of childhood neglect is understudied [4, 5].

Childhood neglect, a form of childhood maltreatment, refers to a situation in which a child's emotional and/or physical needs are not met (e.g., soothing the child when distressed and provision of healthy nutrition [6]). Child neglect can include both minor, isolated incidents and more severe, persistent patterns of failure. This suggests that experiences of neglect exist along a continuum [7], potentially leading to serious health problems that may persist into adulthood [8], including the manifestation of depressive symptoms (for a meta-analysis, see [3]).

The association between childhood neglect and depressive symptoms might be explained by Young's theory of early maladaptive schemas (EMSs) [9]. EMSs are conceptualized as cognitive and emotional patterns that develop during childhood and adolescence in response to unmet emotional needs, trauma, or adverse experiences. Young proposed 18 core EMS, each reflecting a maladaptive theme or pattern that can impact an individual's overall psychological well-being. Emotional and physical neglect have been linked to the development of schemas such as “social isolation,” “emotional deprivation,” and “mistrust abuse” (for a meta-analysis, see [10]), which shape how individuals perceive themselves and their relationships with others. For example, a caretaker who consistently does not meet the emotional needs of a child might give rise to the development of a “social isolation” schema, which is characterized by the feeling of being isolated, different from others and not a part of any group or community. Activation of this schema may hinder individuals from connecting with others and contributing to feelings of loneliness and sadness, which are common components of depression. EMSs in general, and the schemas revolving around emotional deprivation, isolation, and mistrust in particular, are cognitive risk factors for depression [11, 12]. Hence, childhood neglect may lead to the development of EMS, thereby contributing to the emergence and persistence of depressive symptoms.

Despite its high prevalence, research on the associations between neglect and depression has been “neglected” [5, 13]. Most research on childhood maltreatment focused on either childhood abuse only [5] or childhood maltreatment as a broad overall category [14–16]. However, childhood abuse and neglect may have different outcomes on individuals due to the different nature of these experiences. While childhood abuse is defined as an act of commission (i.e., intentional acts of harm or abuse toward a child), childhood neglect is defined as an act of omission (i.e., the failure or omission to provide adequate care, supervision, or support to a child [17]). Childhood abuse often involves single incidents and may also be committed by noncaregivers, while neglect mostly involves chronic situations of neglect by caregivers [18, 19]. Childhood abuse involves direct acts of harm or violence, which may cause fearful arousal and distrust [20], which can lead to anxiety or post-traumatic stress disorder (PTSD) in survivors [21, 22]. Individuals who have experienced abuse may have learned aggressive or maladaptive coping behaviors, such as aggression, defiance, or conduct problems, which may lead to an increase in externalizing behaviors [23]. For example, Colin and colleagues [24] found a positive association between childhood abuse and externalizing behaviors in immigrated high school students. Neglect, on the other hand, may cause feelings of abandonment, low self-esteem, and difficulties with attachment and trust, which may result in symptoms of depression [25–27]. Neglected individuals may exhibit internalizing behaviors, such as social withdrawal, passivity, or difficulties forming relationships, as a result of feeling unimportant or disregarded [26]. For example, a study by VanMeter and colleagues [23] found that childhood emotional neglect lead to depressive symptoms during adolescence. There are also a few studies who found associations between childhood abuse and internalizing symptoms (for a meta-analysis, see [3]) as well as studies on childhood neglect and externalizing symptoms [19]. Emotional neglect is the most commonly reported form of childhood maltreatment in depressed individuals [28], and emotional and physical neglect also predict a less favorable course of depression than childhood abuse [29]. The present study will focus on childhood neglect while controlling for the presence of childhood abuse.

To design targeted interventions that promote the mental health of individuals who experienced childhood neglect, it is important to understand the (maladaptive) coping mechanisms they use when dealing with challenges. In previous studies, coping has been shown to mediate the associations between childhood abuse/neglect and depressive symptoms [15, 16]. Coping has been defined as cognitive and behavioral efforts to manage stressful situations [30]. An avoidant coping style, characterized by cognitive and behavioral efforts to avoid dealing directly with a stressor, such as substance use, behavioral disengagement, or self-blame [31], has been consistently linked to depressive symptoms [32].

Individuals who experienced childhood neglect may develop coping mechanisms aimed at avoiding the emotional distress associated with neglect [33]. While these avoidant coping behaviors may have initially helped children to distance themselves from a stressful situation, they prove to be ineffective in managing negative emotions over time [15]. Developmental psychological theories suggest that in early childhood, children learn how to cope with stressful situations through interaction with their caregivers, including role modeling [34], direct communication [35], and expressions of warmth and support [33]. When children are neglected during this sensitive developmental period and parents lack involvement and responsiveness [36], children fail to acquire adaptive coping behaviors, such as seeking social support or active coping [37]. Instead, neglected children may be more likely to disengage from stressful situations [15] and adapt an avoidant coping style.

Studies have found associations between childhood maltreatment and avoidant coping behaviors, such as substance use [38], behavioral disengagement [15], self-blame [39], or avoidant emotion-focused coping in general [16]. Arslan [40] discovered that avoidant coping behaviors fully mediated the association between childhood maltreatment and mental health. Three of the above-mentioned studies only focused on childhood maltreatment as an overall category [15, 16, 40], while only one study specifically focused on childhood neglect [39]. In the present study, we aim to contribute to the existing literature by specifically focusing on the association between childhood neglect and avoidant coping behaviors.

The COVID-19 pandemic introduced multiple stressors, including self-isolation, curfews, unemployment, financial strain, and COVID-19 infection risk. These factors created a highly suitable context to investigate the associations between childhood neglect, coping, and depressive symptoms. The pandemic intensified mental health challenges and heightened anxiety and depression globally [41], making it essential to understand its specific impact on individuals with a history of childhood neglect. To manage the multitude of stressors and maintain mental health, functional coping strategies were essential for individuals to navigate the complex emotional challenges presented by the pandemic [42]. However, the restrictions posed by governments during the pandemic limited access to certain coping strategies, such as social support networks and professional mental health services, while also inadvertedly supporting reliance on potentially maladaptive coping measures, such as behavioral disengagement and substance use. This isolation not only hindered the use of adaptive coping strategies but also inadvertently encouraged reliance on maladaptive coping strategies, such as behavioral disengagement and substance use.

According to the stress sensitization hypothesis, childhood abuse and neglect make individuals more vulnerable to later stressors, which in turn increases their risk for mental health problems, such as depression [43]. Previous studies have linked the pandemic with higher levels of depressive symptoms in individuals who experienced childhood abuse and neglect [14]. However, research specifically focusing on childhood neglect is limited. Notably, only one study identified a positive association between childhood neglect and depressive symptoms during the pandemic in a sample of the Chinese general population [44]. To our knowledge, no study has yet investigated the specific effect of childhood neglect on depressive symptoms within a sample of the German general population during the COVID-19 pandemic. Furthermore, while existing research has established that self-blame mediates the relationship between childhood maltreatment and depressive symptoms in a sample of the Greek general population during the pandemic [14], the specific coping behaviors of individuals with a history of childhood neglect in Germany remain unexplored. Given the significant impact of childhood neglect on individuals' quality of life, it is crucial to investigate these associations further.

**Figure 1:**
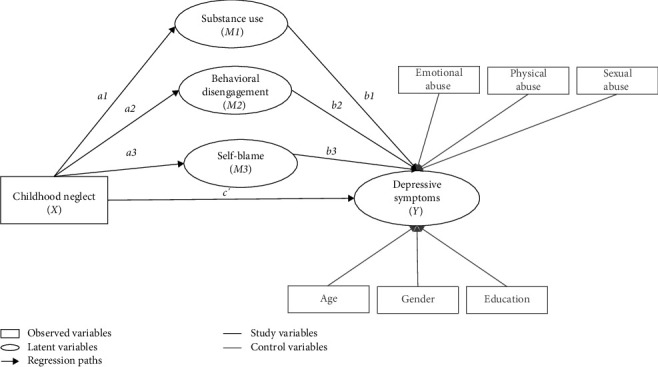
A conceptual diagram of the parallel multiple mediation model.

The present study aims to address this gap by examining how childhood neglect is related to depressive symptoms during the COVID-19 pandemic. Specifically, we investigate whether avoidant coping behaviors, including behavioral disengagement, substance use, and self-blame, mediate this association (Figure 1). We hypothesized that childhood neglect is positively associated with depressive symptoms (H1) and that the relationship between childhood neglect and depressive symptoms is mediated by behavioral disengagement, substance use, and self-blame (H2). Due to the high co-occurrence of maltreatment types [45], the effect of childhood neglect on depressive symptoms may be misestimated if childhood abuse is not considered in the analysis [46]. Therefore, we controlled for childhood abuse (emotional, physical, and sexual) in our study.

## 2. Methods

### 2.1. Design, Sample, and Procedure

This cross-sectional study is a secondary analysis of data obtained from a larger research project, the ADJUST study [47]. The aim of this research project was to determine stressors, coping, and symptoms of adjustment disorder in the course of the COVID-19 pandemic. This study used the German dataset that comprised *N* = 2245 participants from the general population. The sample included both males and females (70.2%), with an overall mean age of 41.1 years (*SD* = 12.5), and a range from 18 to 82 years. Further demographic characteristics, including education level, monthly income, work situation, and experiences of childhood neglect and abuse, were systematically assessed and are detailed in the Results section and Table 1. The inclusion criteria were (1) at least 18 years of age, (2) ability to read and write in German, and (3) written informed consent to participate in the study. Participation in the study was voluntary and participants did not receive a reward. Data collection occurred between June and September 2020 in Germany. The survey was actively promoted through leisure and interest groups (e.g., sports clubs, fitness studios, and theater groups), organizations (e.g., university press departments and companies), newsletters, and social platforms (e.g., Facebook, Twitter, and Instagram). Participants were informed about the study via the online survey platform *Limesurvey* (LimeSurvey GmbH, version 3.22). Only after accepting the data protection policies and giving electronic consent to participate was given, participants were able to proceed to the online questionnaire. Respondent privacy was ensured by complying with the General Data Protection Regulation (DSGVO) and using pseudonymization. To ensure data integrity and reduce the risk of fraudulent responses, we recorded participants' IP addresses during the online survey. This measure helps identify duplicate responses and enhances the validity of the data collected.

### 2.2. Measures

#### 2.2.1. Depressive Symptoms

We assessed depressive symptoms using the validated Patient Health Questionnaire (PHQ-9) [48]. The PHQ-9 is a self-report questionnaire assessing the frequency of experiencing depressive symptoms (e.g., negative thoughts, fatigue, and loss of interest) over the past 2 weeks on nine 4-point scaled items (0 = “not at all” to 3 = “nearly every day”). A total score can be derived by summarizing all items, with cutoff categories indicating mild (5–9), moderate (10–14), moderately severe (15–19), or severe (≥20) depression. Probable depression is indicated by a cutoff score of 10 [49]. The PHQ-9 is a widely recognized and validated tool for depression screening, with previous studies demonstrating its high reliability, as well as criterion and construct validity [50, 51] also in German samples [52].

#### 2.2.2. Childhood Neglect

Childhood neglect was assessed using two items of the German version of the Adverse Childhood Experience Questionnaire (ACE-D) [53]. Respondents were asked (“yes” vs. “no”) whether they experienced two different types of ACEs before the age of 19 years (emotional and physical neglect). A new variable was created to indicate whether participants experienced one (or both) types of neglect (coded as 1) or none (coded as 0). The ACE-D is a reliable and valid screening instrument for the retrospective assessment of stressful experiences in childhood and adolescence [54].

#### 2.2.3. Avoidant Coping

To assess the coping behaviors of participants, the validated Brief Coping Orientation to Problems Experienced (COPE) [55] was used. With two items each, the subscale behavioral disengagement, substance use, and self-blame were rated on a 4-point-Likert scale (0 = “I have not been doing this at all” to 3 = “I”ve been doing this a lot”). The Brief COPE is an established instrument and has been validated in various clinical and nonclinical populations [56].

#### 2.2.4. Control Variables

Variables that have been found to correlate with depressive symptoms [57] were included as control variables. We assessed sociodemographic variables, including age, gender, and the highest level of education (for more details, see [58]). Childhood abuse was measured with the ACE Questionnaire [59], where respondents had to indicate (“yes” vs. “no”) whether they experienced each type of abuse before the age of 19.

### 2.3. Statistical Analyses

A two-step analytic approach was followed by first estimating the measurement models using CFA (step 1) and then using structural equation modeling (SEM) for the main analyses (step 2). In step 1, we assessed the model fit of the measurement model (Supporting Information 1: Figure S1) using the fit indices *chi-square* (*χ*^2^; *p* > 0.05) in relation to the *degrees of freedom* (*df*), *comparative fit index* (CFI; ≥0.95), *Tucker–Lewis Index* (TLI; ≥0.95), *root mean square error of approximation* (RMSEA; ≤0.06), and *standardized root mean residual* (SRMR; <0.008). Interpretation of these indices followed the criteria established by Hu and Bentler [60].

In step 2, the hypotheses were tested using a path model. To test the first hypothesis (H1), which postulates a direct relationship between the independent variable *childhood neglect* and the dependent variable *depressive symptoms*, the latent variable *depressive symptoms* was regressed on the latent variable *childhood neglect*. Furthermore, the variables *emotional*, *physical*, and *sexual abuse* and *age*, *gender*, and *education* were added as control variables. To test the second hypothesis (H2), instead of fitting three linear regression models per mediator, we tested a simultaneously parallel multiple mediation model. We added three coping behaviors (*substance use*, *behavioral disengagement*, and *self-blame*) as mediators into the model described above. This approach poses an advantage compared to three single mediation models as it allows for the assessment of the total indirect effect of a set of mediators as well as the specific indirect effects of each mediator. As recommended by Preacher and Hayes [61], bias-corrected 95% confidence intervals (BC 95% CI) were generated on the basis of 2000 bootstrap samples for specific indirect effects. If the BC 95% CI does not contain zero, the indirect effect significantly differs from zero [62]. The weighted least squares mean and variance adjusted (WLSMV) estimator was used as it does not assume normality and is recommended for modeling categorical data [63]. All analyses were conducted with MPlus (version 8.8) for macOS.

## 3. Results

### 3.1. Sample Characteristics

We recruited *N* = 2744 German participants, of whom 2245 were retained for analysis as they completed the dependent variable, resulting in a mean age of *M* = 41.1 years (*SD* = 12.5). The sample was predominantly female and highly educated but varied in age and income (Table 1). Of these participants, *n* = 501 indicated that they have experienced neglect (emotional and/or physical) before the age of 19 (frequencies of each type of abuse/neglect can be found in Supporting Information 2: Table S1). Of the 501 participants with childhood neglect, 482 participants only experienced emotional neglect, 12 only physical neglect, and 57 both types of neglect. Of all participants, 300 participants experienced neglect and abuse in their childhood, 227 participants only experienced at least one type of abuse, and 203 participants experienced at least one type of neglect (Supporting Information 3: Table S2).

Intercorrelations between study variables can be found in Supporting Information 4: Table S3. The correlations between depressive symptoms and childhood neglect were positive and significant (*r* = 0.32, *p* < 0.01) and higher than the correlations between depressive symptoms and the control variables emotional abuse, physical abuse, and sexual abuse (*r* = 0.24, *p* < 0.01; *r* = 0.12, *p* < 0.01; and *r* = 0.16, *p* < 0.01, respectively). Depressive symptoms positively and significantly correlated with the mediators substance use, behavioral disengagement, and self-blame (*r* = 0.29, *p* < 0.01; *r* = 0.26, *p* < 0.01; and *r* = 0.49, *p* < 0.01, respectively). The intercorrelations between the independent variable neglect and the three mediators substance use, behavioral disengagement, and self-blame were also positive and significant (*r* = 0.14, *p* < 0.01; *r* = 0.13, *p* < 0.01; and *r* = 0.24, *p* < 0.01, respectively). The correlations between emotional abuse, physical abuse, and sexual abuse and the mediators substance use (*r* = 0.08, *p* < 0.01; *r* = 0.06, *p* < 0.01; and *r* = 0.05, *p* < 0.05, respectively) and self-blame (*r* = 0.15, *p* < 0.01; *r* = 0.07, *p* < 0.01; and *r* = 0.08, *p* < 0.01, respectively) were positive and significant but lower than the correlations of childhood neglect and the mediators. The association between physical abuse and behavioral disengagement was nonsignificant (*r* = 0.02, *p* > 0.05), while emotional and sexual abuse showed positive and significant but low correlations with behavioral disengagement (*r* = 0.08, *p* < 0.01, and *r* = 0.06, *p* < 0.01, respectively).

### 3.2. Measurement Model

The measurement model including the three mediators showed acceptable fit (*χ*^2^ = 1270.634, *df* = 182, *p* < 0.01, CFI = 0.97, TLI = 0.97, RMSEA = 0.05, and SRMR = 0.07; for factor loadings of the model, see Supporting Information 5: Table S4).

### 3.3. Direct Influences

**Figure 2:**
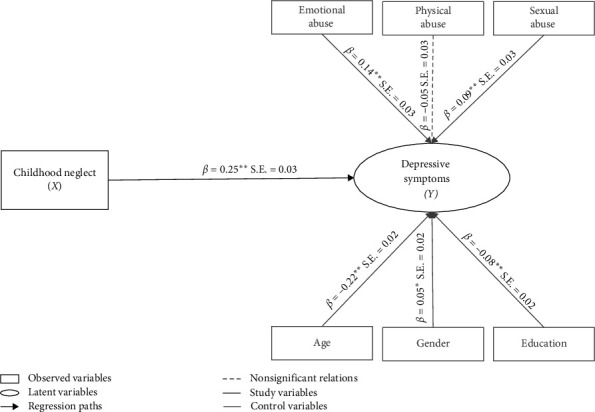
Standardized beta coefficients of the main effect. *Notes: N* = 2245. *p*^⁣*∗*^  < 0.05, two-tailed. *p*^⁣*∗∗*^  < 0.01, two-tailed.

**Figure 3:**
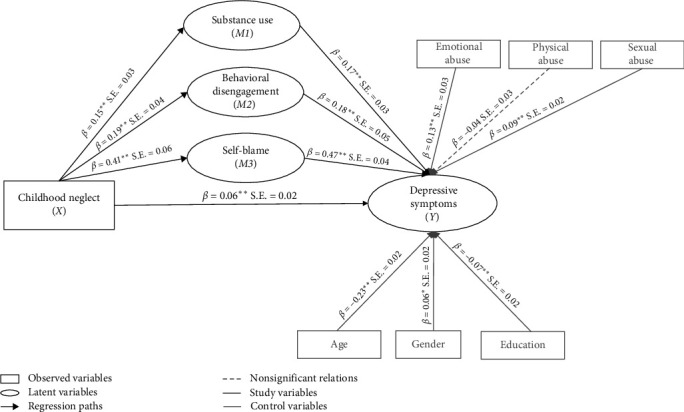
Standardized beta coefficients of the mediation model. *Notes: N* = 2245. *p*^⁣*∗*^  < 0.05, two-tailed. *p*^⁣*∗∗*^  < 0.01, two-tailed.

In the structural model of the main effect (Figure 2), the direct effect (*c* path) between childhood neglect and depressive symptoms was positive and statistically significant (*β* = 0.25, S.E. = 0.03, *p* < 0.01) with no mediators in the model but controlling for age, gender, education, childhood physical, emotional, and sexual abuse, thereby confirming Hypothesis 1 (H1). When the effects of the mediators were included in the model (Figure 3), the direct path (*c′* path) between childhood neglect and depressive symptoms was lower but still significant (*β* = 0.06, S.E. = 0.02, *p* < 0.01). Furthermore, our three proposed mediators were regressed onto childhood neglect (*a* paths). Analyses showed that childhood neglect was significantly associated with increased substance use (*β* = 0.15, S.E. = 0.03, *p* < 0.01), behavioral disengagement (*β* = 0.19, S.E. = 0.04, *p* < 0.01), and self-blame (*β* = 0.41, S.E. = 0.06, *p* < 0.01). Next, we tested associations between all proposed mediators and depressive symptoms (*b* paths). Results indicated that higher levels of substance use (*β* = 0.17, S.E. = 0.03, *p* < 0.01), behavioral disengagement (*β* = 0.18, S.E. = 0.05, *p* < 0.01), and self-blame (*β* = 0.47, S.E. = 0.04, *p* < 0.01) were significantly associated with higher levels of depressive symptoms. The associations between depressive symptoms and the control variables emotional abuse (*β* = 0.13, S.E. = 0.03, *p* < 0.01) and sexual abuse (*β* = 0.09, S.E. = 0.02, *p* < 0.01) were positive and significant. There was no significant association between depressive symptoms and physical abuse (*β* = −0.04, S.E. = 0.03, *p* > 0.05).

### 3.4. Indirect Influences

The mediation model indicated significant indirect effects of childhood neglect on depressive symptoms via the mediators substance use (*b* = 0.05, S.E. = 0.01) (BC 95% CI, 0.03, 0.08), behavioral disengagement (*b* = 0.06, S.E. = 0.02) (BC 95% CI, 0.03, 0.12), and self-blame (*b* = 0.23, S.E. = 0.03) (BC 95% CI, 0.17, 0.30), in the expected direction (Table 2), thereby confirming Hypothesis 2 (H2). To test if one of the mediators differed significantly from the other in terms of magnitude, pairwise contrasts of the indirect effects were conducted (Table 2). Results showed significant contrasts between substance use and self-blame (BC 95% CI, −0.12, −0.01). The contrast between substance use and behavioral disengagement (BC 95% CI, −0.08, 0.04) and the contrast behavioral disengagement and self-blame (BC 95% CI, −0.11, 0.00) were not significant. Hence, the indirect effect of self-blame is significantly higher than substance use but not significantly different from the indirect effect of behavioral disengagement. The results underscore the importance of considering avoidant coping behaviors as key factors influencing the relationship between childhood neglect and depressive symptoms, suggesting that these findings could inform the development of tailored interventions.

## 4. Discussion

The present study is the first to explore the associations between childhood neglect, avoidant coping behaviors, and depressive symptoms during the COVID-19 pandemic in the general population. The findings highlight the importance of childhood neglect and coping behaviors in relation to depressive symptoms. The results indicate that childhood neglect was related to depressive symptoms after adjusting for age, gender, education, and childhood abuse. Since abuse and neglect often co-occur, it is hard to disentangle their unique effects on depressive symptoms. Due to our large sample size, it was possible to distinguish between childhood neglect and abuse.

### 4.1. Associations Between Childhood Neglect and Depressive Symptoms

In line with previous literature both before and during [44] the COVID-19 pandemic, our study confirmed a significant association between childhood neglect and depressive symptoms during the COVID-19 pandemic. The present study's findings highlight the sensitivity of neglected individuals to life stressors and vulnerability to mental health problems [43, 64, 65], suggesting that they represent a significant risk group for mental health issues, particularly during stressful periods. The similarities between the pandemic context and childhood neglect, such as social isolation, and a sense of insecurity about the future may activate memories of past adverse experiences and contribute to the intensification of mental health difficulties among neglected individuals [65]. It is, however, important to note that this knowledge derived from the context of the COVID-19 pandemic can also be applied to other stressor contexts.

Previous studies often focused on childhood maltreatment as an overall category and found positive associations between maltreatment and depressive symptoms [66]. By differentiating between childhood abuse and neglect in our analyses, we aimed to disentangle their distinct effects on depressive symptoms, recognizing the unique emotional and psychological consequences of each. Our findings showed that associations between abuse types and depressive symptoms were weaker than those for neglect, with physical abuse showing no significant relationship. This suggests a qualitative difference in their effects on mental health, with abuse often leading to overt emotional trauma such as anxiety, fear, or PTSD [21, 22] and externalizing behaviors as a result of learned aggressive coping strategies [23]. In contrast, neglect may result in feelings of abandonment and depression [4, 67] and internalizing behaviors and struggle with social withdrawal and relationship difficulties due to feeling unimportant or disregarded [19]. Given these qualitative differences in the effects of abuse and neglect, we controlled for the presence of childhood abuse (emotional, physical, and sexual) in our analyses.

Emotional and sexual abuse was also positively associated with depressive symptoms, while physical abuse was not. Emotional abuse, which involves consistent patterns of humiliation, rejection, or invalidation, can lead to internalizing symptoms, such as depression [68]. Sexual abuse can induce feelings of guilt and shame [69], contributing to depressive symptoms. Conversely, physical abuse might primarily manifest in externalizing behaviors, including aggressive or disruptive behaviors [70], as witnessing violence may result in a justification of aggressive behaviors [71]. A recent study did not find a positive association between physical abuse and depression [70], while others report a weaker relationship compared to other forms of childhood maltreatment [72, 73]. Discrepancies may arise from a failure to control for the presence of childhood neglect [72, 74] or variations in the definitions of physical abuse [75]. These findings highlight the importance of differentiating between and controlling for the presence of different types of childhood maltreatment.

### 4.2. The Mediating Role of Avoidant Coping Behaviors

In this study, we also examined whether the association between childhood neglect and depressive symptoms is mediated by the avoidant coping behaviors substance use, behavioral disengagement, and self-blame. Consistent with our expectations, all three coping behaviors significantly mediated the association between childhood neglect and depressive symptoms. These findings underscore the importance of considering avoidant coping behaviors as key factors in understanding the consequences of childhood neglect on mental health, suggesting that these findings could inform the development of tailored interventions. This is in line with previous research on childhood maltreatment that found associations between childhood maltreatment and substance use [38, 76], behavioral disengagement [15], self-blame [39], or avoidant emotion-focused coping in general [16].

Contrast analyses showed that the indirect effect of self-blame was significantly stronger than the indirect effects of substance use. This suggests that self-blame as a coping mechanism may more strongly impact on the relationship between childhood neglect and depressive symptoms. According to Tanzer et al. [39], neglected individuals might have developed a tendency to blame themselves for the absence of social support, leading to the adoption of more general self-blame strategies. These strategies can hinder the ability to integrate and evaluate new information, giving rise to a vicious cycle where depressed individuals are prone to processing information in a dysfunctional manner. Consequently, this cycle may reinforce maladaptive patterns of thinking and behavior.

Investigating which mechanisms best explain the path from childhood neglect to depressive symptoms yields valuable information for future interventions. Additional research on coping behaviors that might reduce the association between childhood neglect and depressive symptoms is needed to determine adaptive coping behaviors for neglected individuals. Since adults who experienced neglect or abuse in their childhood show significantly lower levels of social support compared to matched controls [77], social support coping could be an important coping behavior for neglected individuals. Studies before the pandemic indicate that social support could reduce the effects of childhood maltreatment on mental health problems [77–79]. Furthermore, a study investigating pregnant women with a history of childhood neglect discovered that social support mediated the association between neglect and distress [46]. It is important to note that the results might have differed in a nonpandemic context, due to the availability of other coping resources, such as social support. Furthermore, the pandemic restrictions fostered isolation, making it more convenient for individuals to rely on avoidant coping strategies. However, the knowledge derived from this study may be applied to other stressor contexts, as individuals with a history of neglect may react similarly to various life stressors.

### 4.3. Strengths and Limitations

This study has various strengths, such as the use of validated scales (Brief COPE [55] and PHQ-9 [48]) and the large sample size, including a large sub group that only experienced neglect (and no abuse), making it possible to differentiate between the effects of neglect and abuse. Nevertheless, it is important to also consider the limitations of this study. The sample is not representative of the German general population, as there is an overrepresentation of females and high-educated individuals and only participants with Internet access were able to participate in the study. The gender distribution might have led to an overestimation of the effect since women are generally more likely than men to report higher levels of depressive symptoms [80]. The high level of education, on the other hand, might have represented a protective factor against [81], thereby potentially contributing to an underestimation of the effect of childhood neglect. Furthermore, individuals who were highly affected (e.g., severely depressed) might have been unlikely to fill in the survey. Furthermore, although we controlled for age, gender, and education level in our analyses, we did not account for other psychiatric conditions, which may limit the generalizability of our findings. The study solely relied on self-report measures, which are susceptible to social desirability bias [82]. The variable *neglect* did not specify which type of neglect participants experienced (emotional or physical neglect) because the number of individuals who experienced physical neglect was small (*n* = 69; 2.72%) and only *n* = 12 (0.53%) reported physical neglect and no emotional neglect in their childhood. This prevalence is in contrast with a study that investigated prevalence rates of childhood maltreatment in the German general population and found a prevalence of physical neglect of 22.5% [1]. This divergence in prevalence rates could be due to our sample not being representative of the German general population (see above) or the use of the Childhood Trauma Questionnaire (CTQ) [83] versus the ACE-D Questionnaire [53] in our study.

Since this study adopts a cross-sectional design, it is important to acknowledge that the direction of the mediation is based on the assumption that childhood experiences are past events, while depressive symptoms and coping behaviors are considered current experiences. However, alternative directions of causality cannot be ruled out. It could also be that higher levels of depressive symptoms lead to the increased use of substance use, behavioral disengagement and self-blame, or recurring memories of childhood neglect. These coping behaviors could be both risk factors or symptoms of depression [84–86]. Hence, no causal inferences can be drawn from the findings. Moreover, the reliance on a cross-sectional design may limit our understanding of the temporal dynamics between childhood neglect and depressive symptoms, potentially obscuring the effects of intervening variables over time. Future research might focus on longitudinal studies to test the directionality of coping behaviors and depressive symptoms in individuals who experienced childhood neglect.

### 4.4. Implications

Despite the high prevalence of childhood neglect, most research focused on childhood maltreatment as an overall category or only on abuse. The present study specifically investigated the effect of childhood neglect on depressive symptoms and coping behaviors during the COVID-19 pandemic while controlling for childhood abuse. Recognizing and understanding the unique impacts of childhood neglect on mental health are crucial for developing tailored interventions to support individuals who experienced childhood neglect, especially during stressful times, such as the COVID-19 pandemic. Given the significant association identified, it is imperative for clinical practitioners to integrate specific screening tools for childhood neglect into their assessments. Furthermore, our findings underscore the role of avoidant coping in explaining the association between childhood neglect and depressive symptoms. To address this in practice, we recommend that psychological interventions incorporate strategies to enhance adaptive coping skills and reduce avoidant behaviors among individuals with a history of childhood neglect. On a broader scale, public health policies could raise public awareness about the signs of childhood neglect and provide resources for early intervention.

## 5. Conclusion

Childhood neglect was positively associated with depressive symptoms after adjusting for childhood abuse. Survivors of childhood neglect represent a relevant risk group for depression during severe community stressors such as the COVID-19 pandemic. Individuals with a history of childhood neglect used more avoidant coping during the pandemic, including substance use, behavioral disengagement, and self-blame, which in turn increased their depressive symptoms. Targeting these avoidant coping behaviors in survivors of neglect by psychosocial interventions may help to reduce depressive symptoms.

## Figures and Tables

**Table 1:** Sociodemographic characteristics of the sample (*N* = 2245).

Characteristics	
Age (years)	*M* (*SD*)
Mean	41.1 (12.5)
Range	18–82
Gender	*n* (%)
Male	661 (29.4)
Female	1575 (70.2)
Diverse	9 (0.4)
Education (highest degree)	
<10 years schooling	7 (0.3)
≥10 years schooling	280 (12.5)
Completed vocational studies	785 (35.0)
Completed studies	1173 (52.2)
Monthly household income (net)^a^	
Very low (<500 €)	79 (3.7)
Low (500–<1000 €)	140 (6.5)
Middle (1000–<3000 €)	867 (40.3)
High (≥3000 €)	1065 (49.5)
Training/work situation^b^	
(Vocational) studies	348 (15.5)
Employed full-time	1119 (49.8)
Self-employed	85 (3.8)
Freelancer	62 (2.8)
Retired	95 (4.2)
Seeking work	51 (2.3)
Other	138 (6.1)

*Note: N* = 2245.

^a^ *n* = 2151.

^b^Multiple answers were possible.

**Table 2:** Mediation of the effect of childhood neglect on depressive symptoms through the coping behavior substance use, behavioral disengagement, and self-blame.

	Bootstrapping	
	BC 95% CI	
	Lower	Upper
Indirect effects (unstandardized beta coefficients)		
Substance use^a^	0.03	0.08
Behavioral disengagement^a^	0.03	0.12
Self-blame^a^	0.17	0.30
Total (avoidant coping)^a^	0.26	0.42
Contrasts of indirect effects (unstandardized beta coefficients)		
Substance use vs. behavioral disengagement	−0.08	0.04
Substance use vs. self-blame^a^	−0.12	−0.01
Behavioral disengagement vs. self-blame	−0.11	0.00

*Note: N* = 2245; bias-corrected 95% confidence intervals (BC 95% CI); 2000 bootstrap samples.

^a^Significant (zero not contained in CI).

## Data Availability

The detailed sociodemographic information of the dataset does not fully protect the anonymity of the respondents. For this reason, the entire dataset cannot be made publicly available. However, excerpts of the data on a higher aggregation level can be provided upon justified request by the last author.

## Supporting Information

Supporting Information 1 Figure S1: This figure shows the measurement model of the study, illustrating how the latent variables (e.g., coping behaviors and depressive symptoms) were measured. While the model is also described in the main text, the figure provides a visual representation that aids in understanding the model structure.

Supporting Information 2 Table S1: This table presents the frequencies of the five subtypes of adverse childhood experiences observed in our sample. It provides detailed demographic information by showing the distribution of different types of adverse childhood experiences, which is crucial for understanding the sample's characteristics.

Supporting Information 3 Table S2: This table presents the cross-tabulated frequencies of childhood abuse and neglect, showing how many participants experienced only neglect, only abuse, or both. This differentiation is essential as we aimed to analyze the specific effect of childhood neglect on depressive symptoms, controlling for the presence of childhood abuse.

Supporting Information 4 Table S3: This table shows the descriptive statistics and correlations among the study variables. This table provides a comprehensive overview of the relationships between key variables in the study and as well as the reliability and validity of the measures used.

Supporting Information 5 Table S4: This table shows the factor loadings of the measurement models. Although the main text mentions that the model fit was acceptable, this table provides the exact values of the factor loadings, allowing for a more detailed examination of the measurement model's validity.
