# Supplementary material for: Associations Between Childhood Neglect and Depressive Symptoms: The Mediating Effect of Avoidant Coping
Source: Depress Anxiety. 2024 Nov 30;2024:9959689. doi: 10.1155/da/9959689 (PMC11918893; doi:10.1155/da/9959689)
Supplement: Supporting Information 1 — Figure S1: This figure shows the measurement model of the study, illustrating how the latent variables (e.g., coping behaviors and depressive symptoms) were measured. While the model is also described in the main text, the figure provides a visual representation that aids in understanding the model structure. [file 9959689.f1.docx]

**Figure S1**

*The Measurement Model*


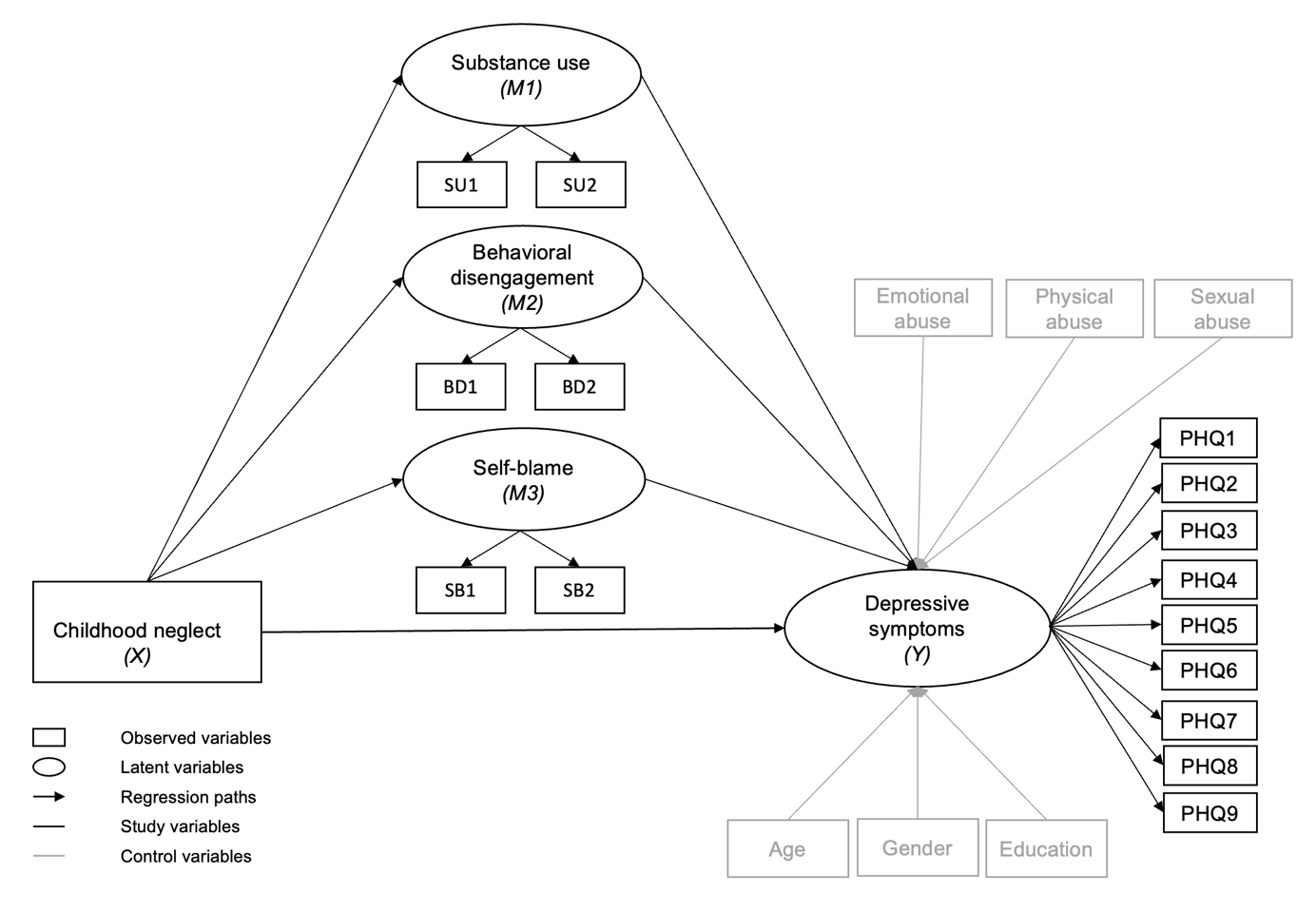


*Note.* The two categories physical and emotional neglect were collapsed into one dichotomous category named childhood neglect.
