# Supplementary material for: Associations Between Childhood Neglect and Depressive Symptoms: The Mediating Effect of Avoidant Coping
Source: Depress Anxiety. 2024 Nov 30;2024:9959689. doi: 10.1155/da/9959689 (PMC11918893; doi:10.1155/da/9959689)
Supplement: Supporting Information 2 — Table S1: This table presents the frequencies of the five subtypes of adverse childhood experiences observed in our sample. It provides detailed demographic information by showing the distribution of different types of adverse childhood experiences, which is crucial for understanding the sample's characteristics. [file 9959689.f2.docx]

**Table S1**

*Frequencies of Adverse Childhood Experiences (ACE-D)*

| Type of adverse childhood experience | *n* (%) |
| --- | --- |
| Emotional neglect | 489 (21.8) |
| Physical neglect | 61 (2.7) |
| Emotional abuse | 368 (16.4) |
| Physical abuse | 191 (8.5) |
| Sexual abuse | 227 (10.1) |

*Notes.* *N* = 2245. Different types of adverse childhood experiences can co-occur.
