# Supplementary material for: Associations Between Childhood Neglect and Depressive Symptoms: The Mediating Effect of Avoidant Coping
Source: Depress Anxiety. 2024 Nov 30;2024:9959689. doi: 10.1155/da/9959689 (PMC11918893; doi:10.1155/da/9959689)
Supplement: Supporting Information 3 — Table S2: This table presents the cross-tabulated frequencies of childhood abuse and neglect, showing how many participants experienced only neglect, only abuse, or both. This differentiation is essential as we aimed to analyze the specific effect of childhood neglect on depressive symptoms, controlling for the presence of childhood abuse. [file 9959689.f3.docx]

**Table S2**

*Cross-Tabulated Frequencies of Child Neglect by Abuse.*

|  |  | Abuse | | | |  |
| --- | --- | --- | --- | --- | --- | --- |
|  |  | No | One type | Two types | Three types | Total |
| Neglect | No | 1517 | 174 | 42 | 11 | 1744 |
|  | One type | 197 | 125 | 106 | 24 | 452 |
|  | Two types | 4 | 16 | 17 | 12 | 49 |
| Total |  | 1718 | 315 | 165 | 47 | 2245 |
