# Supplementary material for: Associations Between Childhood Neglect and Depressive Symptoms: The Mediating Effect of Avoidant Coping
Source: Depress Anxiety. 2024 Nov 30;2024:9959689. doi: 10.1155/da/9959689 (PMC11918893; doi:10.1155/da/9959689)
Supplement: Supporting Information 4 — Table S3: This table shows the descriptive statistics and correlations among the study variables. This table provides a comprehensive overview of the relationships between key variables in the study and as well as the reliability and validity of the measures used. [file 9959689.f4.docx]

Table S3
*Means (M), Standard Deviations (SD), Skewness (SK), Kurtosis (KU), Composite Reliability (CR), and Pearson Correlations between Depression (PHQ-9), Neglect, Substance Use, Behavioral Disengagement, Self-Blame, Age, Gender, and Education*

|  | *M* | *SD* | SK | KU | CR | 1 | 2 | 3 | 4 | 5 | 6 | 7 | 8 | 9 | 10 |
| --- | --- | --- | --- | --- | --- | --- | --- | --- | --- | --- | --- | --- | --- | --- | --- |
| 1. Depressive symptoms (PHQ-9) | 6.60 | 5.02 | 1.01 | .66 | .91 |  |  |  |  |  |  |  |  |  |  |
| 2. Neglect^a^ | - | - | 1.33 | -.23 | - | .32** |  |  |  |  |  |  |  |  |  |
| 3. Substance use | .70 | 1.33 | 2.19 | 4.50 | .92 | .29** | .14** |  |  |  |  |  |  |  |  |
| 4. Behavioral disengagement | .98 | 1.12 | 1.40 | 2.09 | .34 | .26** | .13** | .15** |  |  |  |  |  |  |  |
| 5. Self-blame | .69 | 1.26 | 2.22 | 4.91 | .72 | .49** | .24** | .17** | .19** |  |  |  |  |  |  |
| 6. Age | 41.07 | 12.5 | .22 | -.73 | - | -.21** | -.02 | <.001 | -.06** | -.23** |  |  |  |  |  |
| 7. Gender^a^ | - | - | -.80 | -1.02 | - | .12** | .10** | -.03 | .02 | .11** | -.10** |  |  |  |  |
| 8. Education | 3.39 | .71 | -.78 | -.47 | - | -.21** | -.06** | -.08** | -.08** | -.08** | -.18** | .12** |  |  |  |
| 9. Emotional abuse^a^ | - | - | 1.82 | 1.30 | - | .24** | .51** | .08** | .08** | .15** | .05* | .09** | -.08** |  |  |
| 10. Physical abuse^a^ | - | - | 2.98 | 6.87 | - | .12** | .34** | .06** | .02 | .07** | .06** | .02 | -.07** | .59** |  |
| 11. Sexual abuse^a^ | - | - | 2.65 | 5.02 | - | .16** | .20** | .05* | .06** | .08** | .03 | .13* | -.01 | .20** | .16** |

*Notes. N* = 2245*.* PHQ-9 = sum score of 9 items rated on 4-point Likert scales (0 = ‘not at all’ to 3 = ‘nearly every day’); neglect 0 = no childhood neglect, 1 = physical and/or emotional childhood neglect; Male = 1; Female = 2; Other = 3. Education ranges from 1 (> 10 years schooling) to 4 (complete studies); emotional abuse 0 = no emotional abuse, 1 = emotional abuse; physical abuse 0 = no physical abuse, 1 = physical abuse; sexual abuse 0 = no sexual abuse, 1 = sexual abuse. ^a^point biserial correlation. **p* < .05, ***p* < .01.
