# Supplementary material for: Associations Between Childhood Neglect and Depressive Symptoms: The Mediating Effect of Avoidant Coping
Source: Depress Anxiety. 2024 Nov 30;2024:9959689. doi: 10.1155/da/9959689 (PMC11918893; doi:10.1155/da/9959689)
Supplement: Supporting Information 5 — Table S4: This table shows the factor loadings of the measurement models. Although the main text mentions that the model fit was acceptable, this table provides the exact values of the factor loadings, allowing for a more detailed examination of the measurement model's validity. [file 9959689.f5.docx]

**Table S4**

*Factor Loadings of the Measurement Model*

|  | Factor 1^a^ | Factor 2^a^ | Factor 3^a^ | Factor 4^a^ |
| --- | --- | --- | --- | --- |
| PHQ1 | .73 |  |  |  |
| PHQ2 | .87 |  |  |  |
| PHQ3 | .68 |  |  |  |
| PHQ4 | .83 |  |  |  |
| PHQ5 | .68 |  |  |  |
| PHQ6 | .75 |  |  |  |
| PHQ7 | .76 |  |  |  |
| PHQ8 | .60 |  |  |  |
| PHQ9 | .72 |  |  |  |
| SUB1 |  | .86 |  |  |
| SUB2 |  | .99 |  |  |
| BEHD1 |  |  | .40 |  |
| BEHD2 |  |  | .50 |  |
| SBL1 |  |  |  | .70 |
| SBL2 |  |  |  | .80 |

*Notes. N* = 2245*.* Factor 1 = Depressive symptoms (PHQ-9); Factor 2 = Substance use, Factor 3 = Behavioral disengagement, Factor 4 = Self-blame, PHQ1 – PHQ9 = Items 1-9 of the PHQ-9, SUB1 = Item 1 substance use, SUB2 = Item 2 substance use, BEHD1 = Item 1 behavioral disengagement, BEHD2 = Item 2 behavioral disengagement, SBL1 = Item 1 self-blame, SBL2 = Item 2 self-blame.

^a^ First-order factor.
